# Small cell lung cancer transformation during antitumor therapies: A systematic review

**DOI:** 10.1515/med-2021-0321

**Published:** 2021-08-11

**Authors:** Xing Chai, Xinru Zhang, Wenqian Li, Jin Chai

**Affiliations:** Department of Procurement Center, The Second Hospital of Jilin University, Changchun, 130041, China; Department of Pharmacy, The Second Hospital of Jilin University, Changchun, Jilin, 130041, China; Department of Cancer Center, The First Hospital of Jilin University, Changchun, Jilin, 130021, China; Department of Pharmacy, The Second Hospital of Jilin University, No.218 Ziqiang Street, Nanguan District, Changchun, Jilin, 130041, China

**Keywords:** transformation, NSCLC, SCLC

## Abstract

Lung cancer is the most common cause of cancer-related death. Non-small cell lung cancer (NSCLC) and small cell lung cancer (SCLC) are the two major histological categories of lung cancers. Drug resistance is a great challenge for cancer treatment, and histological transformation from NSCLC to SCLC is one of the mechanisms underlying drug resistance in NSCLC patients. SCLC-transformed patients show combined characteristics of NSCLC and SCLC; however, they lack timely diagnoses and effective treatment strategies. Thus, we reviewed the clinical characteristics of SCLC transformation patients with a literature search to enhance clinical consciousness, diagnosis, and personalized treatment for patients with it.

## 1 Introduction

Lung cancer has the highest mortality rate among all cancer types, and it is classified into two major categories: non-small cell lung cancer (NSCLC) occurs in approximately 85% of cases, while small cell lung cancer (SCLC) accounts for the remaining 15% [1]. Adenocarcinoma and squamous cell carcinoma are the main pathological types of NSCLC, accounting for more than 70% of all cases [2]. Despite the multiple choices of antitumor therapies, the 5-year overall survival (OS) remains poor, ranging from 4 to 17%, with differences in staging and pathology [3,4]. In 2006, the first SCLC transformation was reported in a 45-year old woman who was diagnosed with adenocarcinoma and received targeted therapy, radiotherapy, and chemotherapy before re-biopsy. However, despite the combination therapy of gefitinib and etoposide after transformation, the patient did not have a good survival [5]. Since then, the histological transformation from NSCLC to SCLC has become an increasingly noticeable form of resistance in recent years. Previous studies have shown that the incidence of SCLC transformation in patients with epidermal growth factor receptor-tyrosine kinase inhibitor (EGFR-TKI) resistance was approximately 14%, and further studies showed that SCLC transformation also occurred after other types of resistance [6,7]. However, the clinical consciousness of histological transformation is suboptimal, and the treatments are still controversial. Thus, this review aimed to explore the occurrence, diagnosis, and treatment strategies of SCLC transformation using a literature search to demonstrate the clinical characteristics of patients with SCLC transformation.

## 2 Methods

**Figure 1:**
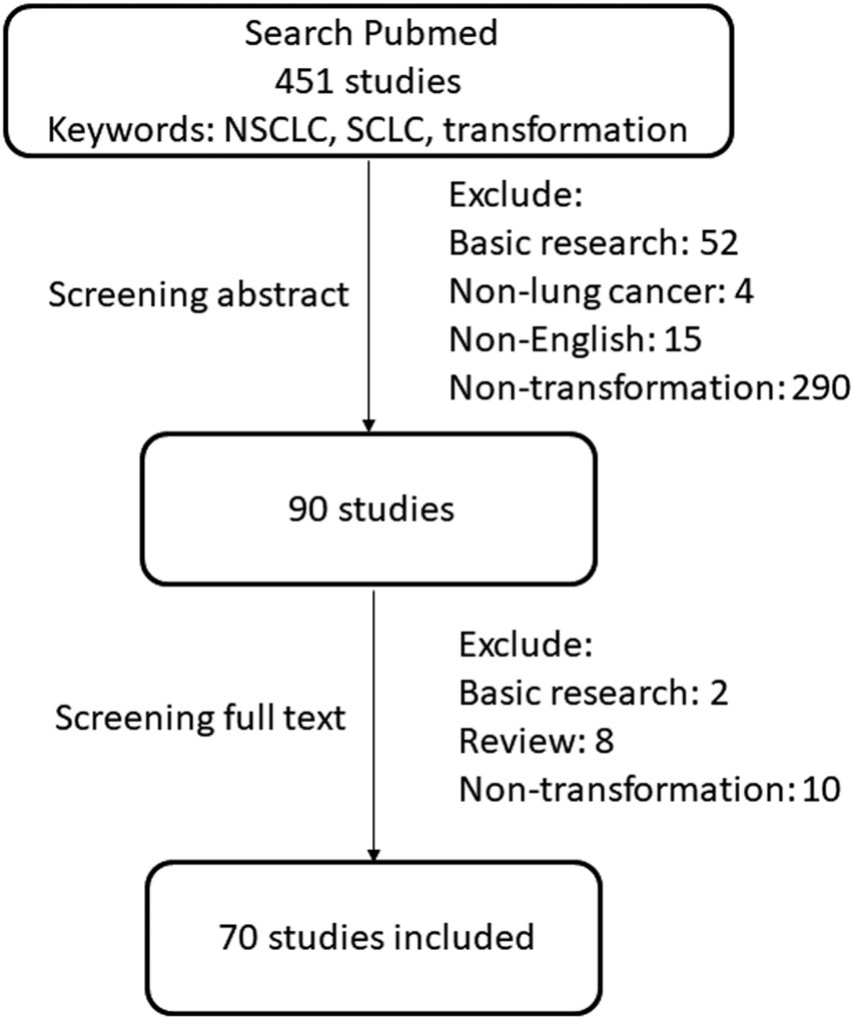
The retrieval flow chart.

We searched the PubMed database for studies published until February 2020. The search keywords included “NSCLC,” “SCLC,” “adenocarcinoma,” “squamous cell carcinoma,” and “transformation.” Two investigators independently searched for and screened the literature. The included studies focused on patients who underwent transformation from NSCLC to SCLC. Clinical cases, retrospective studies, and clinical trials were eligible, and the included studies were published in English. The exclusion criteria included basic research, studies focused on other types of cancer, non-English articles, and review articles. A total of 451 published studies were found, of which 90 studies were left after screening abstracts. Seventy studies were finally included after a further screening of the full texts. The retrieval flow chart is shown in Figure 1.

Among the 70 studies, most were published in Asian countries (45 studies, 64.3%). Despite 132 transformed SCLC patients without specific information in five retrospective studies (summarized in Table 1), 65 studies with 104 patients were included in the following analysis, where we summarized the clinical characteristics of patients with transformed SCLC.

**Table 1:** Characteristics of five retrospective studies

	Patients	Year	Country	Pathologic types	Prognosis	References
1	46	2018	US	30 Adenocarcinoma	mOS: 304 days	[58]
				16 Squamous cell carcinoma		
2	3	2019	Korea	Adenocarcinoma	—	[59]
3	4	2019	France	Adenocarcinoma	—	[60]
4	58	2018	US	Adenocarcinoma	mOS: 10.9 months	[42]
5	21	2017	Korea	Adenocarcinoma	—	[26]

mOS: median overall survival.

## 3 Clinical characteristics of patients with SCLC transformation

### 3.1 Clinical characteristics before transformation

**Figure 2:**
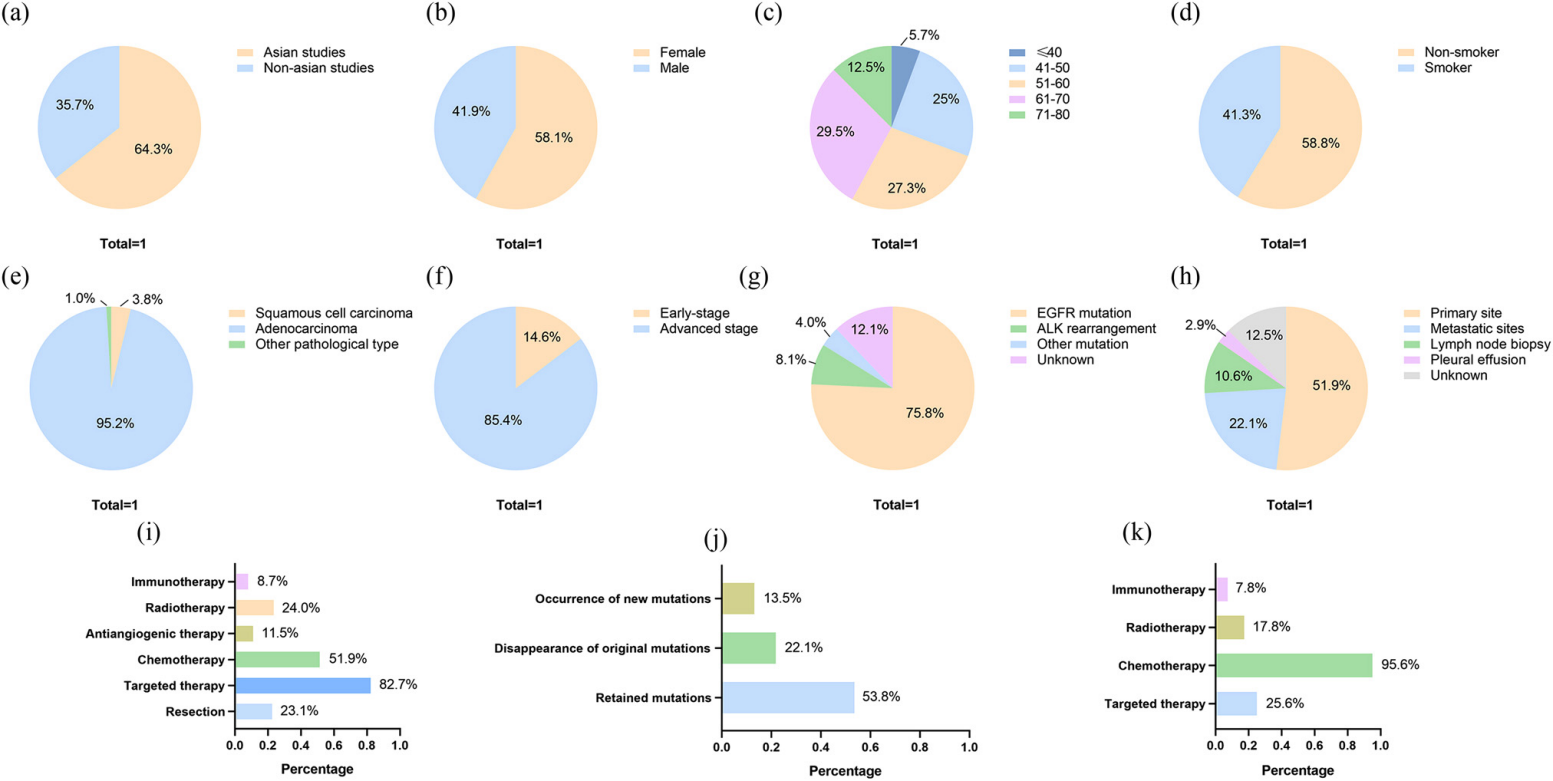
Clinical characteristics of patients. (a) Published countries of studies. (b) Gender information of patients. (c) Age of patients. (d) Smoking history of patients. (e) Pathological type of patients. (f) Stage information of patients. (g) Mutations of patients with adenocarcinoma. (h) Re-biopsy sites of patients. (i) Treatments before transformation. (j) Mutations in re-biopsy. (k) Treatments after transformation.

The clinical characteristics of the patients are summarized in Figure 2. Of the 86 patients with reported gender information, 50 (58.1%) were women and 36 (42%) were men. Transformation mostly occurred in patients aged 61–70 years (26 patients, 29.5%), followed by the 51–60 years group (24 patients, 27.3%) and 41−50 years group (22 patients, 25%). Among the 80 patients with a history of smoking, 33 (41.3%) were previous or current smokers, while 47 (58.8%) were nonsmokers. Five patients were diagnosed with preexisting disease, and three patients had a history of other types of cancer.

Eighty-two patients were diagnosed based on TNM stage information, 70 patients (85.4%) were diagnosed with locally advanced or metastatic NSCLC, while 12 patients (14.6%) were diagnosed with early stage NSCLC. Histologically, the main pathological type was adenocarcinoma (99 patients, 95.2%), and four patients (3.8%) were diagnosed with squamous cell carcinoma. Among the 99 adenocarcinoma patients, epidermal growth factor receptor (EGFR) mutations were detected in 75 patients (75.8%), and ALK rearrangement was found in eight patients (8.1%). In addition, three patients had K-RAS mutations, and one patient had PD-L1 positive NSCLC.

Based on the pathological types and gene mutations, the majority of patients were administered targeted therapy (86 patients, 82.7%) or chemotherapy (54 patients, 51.9%). Besides, 25 patients (24.0%) received radiotherapy, 24 patients (23.1%) underwent resection at the primary site, 12 patients (11.5%) received antiangiogenic therapy, and nine patients (8.7%) received immunotherapy.

### 3.2 Clinical characteristics after transformation

Re-biopsy is necessary for the detection of SCLC transformation. Apart from the 13 patients (12.5%) without a description of the method of re-biopsy, most of the patients (54 patients, 51.9%) underwent re-biopsy at the primary site, 23 patients (22.1%) were diagnosed with transformation through a biopsy at the metastatic sites, 11 patients were diagnosed (10.6%) through lymph node biopsy, and three patients (2.9%) had cytological evidence of pleural effusion. Considering that gene mutations can guide treatment decisions, a second mutation test was also significant. Fifty-six patients (53.8%) were found to have retained mutations during re-biopsy similar to those for the initial diagnosis. The original mutations could not be found in 23 patients (22.1%), and new mutations were observed in 14 patients (13.5%), five of whom were found to have the Thr790Met mutation. In some cases, the disappearance of the original mutation occurs simultaneously with the appearance of a new mutation. For example, it has been reported that a patient showed the disappearance of the original exon 19 mutation and a new L858R and Thr790Met mutation during re-biopsy at the same time [8]. Another patient was found to have reduced expression of programmed cell death ligand 1 (PD-L1) from 30% to less than 1% [9]. In addition, 19 patients were reported to have changes in tumor biomarkers; 14 patients were found to have an increase in neuron-specific enolase (NSE) and eight patients had an increase in prn-gastrin-releasing peptide (Pro-GRP).

Regarding the treatments for SCLC transformation, treatment strategies after diagnosis with transformation were reported for 90 patients. Almost all patients (86 patients, 95.6%) received chemotherapy (mainly platinum and etoposide), which is widely recognized as a sensitive therapy for SCLC. Twenty-three patients (25.6%) underwent targeted therapy according to the results of the gene mutations, 16 patients (17.8%) received radiotherapy, and seven patients (7.8%) received immunotherapy.

## 4 Enhanced awareness and individualized treatment

### 4.1 Explore potential mechanism

Currently, there are two major hypotheses on the mechanism of transformation from NSCLC to SCLC. The first hypothesis suggested that both NSCLC and SCLC components coexisted in tumor tissues, and only a part of them were detected during the initial biopsy, which gradually became dominant after treatment for NSCLC. Some patients were initially diagnosed with combined NSCLC and SCLC without receiving any antitumor treatment, which supports this hypothesis [10]. However, SCLC transformation was also observed in patients who underwent tumor resection with abundant histopathological tissue. In addition, SCLC patients without timely treatment progressed rapidly and had a poor prognosis, and their progression-free survival (PFS) was significantly reduced, which was also contrary to the hypothesis [11].

The second hypothesis was the theory for the transformation from NSCLC to SCLC, in which tumor stem cells would differentiate into NSCLC cells before undergoing SCLC transformation during the process of proliferation under the influence of external factors, such as EGFR-TKI treatment. Studies have demonstrated that epigenetic regulation is associated with changes in cell fate; transdifferentiation is a type of cell lineage plasticity, while mechanistic exploration has not been fully elucidated [12]. Current studies have shown that tumor cells first enter a reversible slow-growing drug-tolerant state, called “drug-tolerant persisters (DTPs),” and sensitivity can be regained after removing external stimuli [13,14,15]. DTPs can further acquire permanent resistance through reprogramming, which is associated with epigenetic changes, transcription factors, key signaling pathways, and complex tumor microenvironments [16,17]. Takagi et al. reported a 70-year-old woman diagnosed with a mixed component of SCLC and adenocarcinoma, with a rare mutation, L861Q, in both parts, which suggested that SCLC and NSCLC were more likely from the same progenitor [18]. In addition, more than half of the patients retained their original mutations after transformation according to our statistics, which also supported the second hypothesis.

The transformation of SCLC was also demonstrated at the molecular level in processes including retinoblastoma 1 gene (RB1) deletion and tumor protein p53 gene (TP53) inactivation [19]. The deletion of RB1 can cause the inhibition of entry into the S-phase of cell cycles, which plays an important role in the tumorigenesis of SCLC [12]. RB1 deletions result in an expression deficiency of retinoblastoma protein, which is one of the significant differences between SCLC and NSCLC [20,21]. TP53 is a tumor suppressor gene, and the inactivation of TP53 leads to an abnormal regulatory effect on cell growth, apoptosis, and DNA repair, which plays an important role in tumor formation [22,23]. Previous studies have shown that TP53-deficient and RB1-deficient cells are involved in the lineage plasticity and neuroendocrine differentiation process, but further evidence is needed to confirm whether there is a causal relationship between these molecular changes and pathological phenotypic changes [24,25]. Currently, complete inactivation of RB1 and TP53 is widely accepted as a predictive biomarker for SCLC transformation [26]. Clinicians should pay more attention to monitoring SCLC transformation during the treatment of NSCLC patients with an inactivated RB1 or P53, and the assessment of RB1 and TP53 status should be performed when the disease progresses, which may unravel a potential SCLC transformation [27]. In addition, other immunohistochemical characteristics of SCLC, including synaptophysin, chromogranin, and CD56, were also found after transformation, which is worthy of further attention [7].

### 4.2 Raise consciousness of transformation

According to the basic clinical characteristics of the current study, it was found that the transformation to SCLC from NSCLC mainly occurred in patients with EGFR-mutant advanced adenocarcinoma with a greater tendency of female Asian patients without a smoking history. The proportion was similar to that of EGFR mutations, which emerged mainly in Asian patients and mostly in adenocarcinoma, women, and never-smokers [4]. SCLC transformation is widely accepted as one of the drug resistance mechanisms of targeted therapy, and previous studies have mainly focused on the transformation of adenocarcinoma; only a few studies have reported patients with squamous cell carcinoma [7]. A possible reason is that alveolar type II cells can differentiate into both EGFR-mutant adenocarcinoma and SCLC, which may contribute to a higher likelihood of SCLC transformation in patients with EGFR-mutant adenocarcinomas [11]. Another possible reason is the high frequency of gene mutations and subsequent resistance to antitumor therapies in patients with adenocarcinoma. Consequently, adenocarcinoma patients with gene mutations were more likely to have re-biopsies and to be diagnosed with SCLC transformation. However, transformation occurred not only in patients with EGFR mutations, but also in patients with ALK rearrangement and other uncommon mutations, and SCLC transformation was also found in patients with wild-type mutations and squamous cell carcinomas [28,29,30]. Thus, the bias associated with clinical practice cannot be ignored. For patients with wild-type mutations or squamous cell carcinomas, re-biopsy should also be considered to detect transformation when the disease progresses.

Subsequently, the transformation of SCLC may occur after various treatment strategies, including different generations of targeted therapies, as well as other antitumor therapies, including chemotherapy, radiotherapy, antiangiogenic therapy, and immunotherapy [31]. When hyper-progressive disease occurs during immunotherapy, SCLC transformation should also not be overlooked, as it may be a cause of resistance to immunotherapy [32]. Thus, despite paying attention to the mechanism of drug resistance after targeted therapy when progressive disease occurs, clinicians should also be aware of transformed SCLC arising after other antitumor therapies. Further studies can explore the potential of SCLC transformation among various NSCLC subsets and patients after different treatment therapies.

Meanwhile, SCLC transformation should be distinguished from combined small cell lung cancer (C-SCLC), which is defined as a subtype of SCLC with an incidence of approximately 2–23% of SCLC [33,34]. Although the potential mechanisms are still unclear, current research suggests that the most common pathological type is squamous cell carcinoma and large cell neuroendocrine carcinoma [35]. C-SCLC was more likely to be found in older men with a long smoking history, which is similar to the incidence of SCLC [36,37].

### 4.3 Strengthen the early diagnostic capacity

Since the majority of patients were preliminarily diagnosed with advanced disease, and disease progression also reduced the likelihood of resection, re-biopsy was necessary for the diagnosis of transformation. In addition to re-biopsy of primary sites, re-biopsies from lymph nodes and metastatic sites could also be conducive to the detection and diagnosis of transformation, and the detection of pleural effusion was also an auxiliary diagnostic method. It was also necessary to carry out gene mutation detection in the re-biopsied tissues. Considering that nearly half of the patients retained mutations, both the disappearance of original mutations and new mutations may occur. Thus, timely detection can facilitate better treatment strategies when drug resistance or unexpected treatment response occurs.

Moreover, patients with SCLC transformation may have multiple tumor lesions with different pathological types. For instance, a non-smoking 76-year-old woman was diagnosed with clinical stage IIIB NSCLC; after chemotherapy and radiotherapy, multiple lung metastases were found 15 months later, and she received targeted therapy until death. At autopsy, nine metastatic lesions were found with different pathological types, including six SCLCs, two NSCLCs, and one retroperitoneal lymph node with combined histology. Furthermore, an EGFR exon 19 deletion mutation was found in all lesions, while the T790M mutation was only found in adenocarcinoma lesions [38]. Therefore, the transformation may be partial and multiple, and the diversity of transformation should not be overlooked, especially for metastatic lesions. This emphasizes the significance of biopsy and suggests a supplemental role of next-generation sequencing (NGS) for ctDNA.

In addition, data showed that some patients had increased tumor biomarkers associated with SCLC, and the most common tumor biomarkers were NSE and Pro-GRP. Although the sensitivity and specificity of tumor biomarkers should be further investigated, considering the simplicity and dynamics of detection, they may provide an early clue for re-biopsy and serve as an auxiliary diagnostic method for SCLC transformation. Thus, in addition to regular imaging evaluations, the monitoring of tumor biomarkers during treatment should also be applied in clinical practice. An elevated NSE or Pro-GRP may be considered for its potential to facilitate transformation.

### 4.4 Proceed active personalized treatment

The prognosis and survival period varied among patients, and most patients who underwent transformation had been treated with multiline treatments. The overall prognosis was poor, and it was associated with the physical condition of the patients. A systematic review that included 39 adenocarcinoma patients showed a median duration of 19 months from the initial diagnosis to SCLC transformation, and a median survival duration of 6 months after the transformed SCLC diagnosis [39]. To improve the prognosis of patients, both the prevention of transformation and personalized treatment after transformation were significant. Effective strategies to prevent transformation include intermittent treatment, combination therapy, and the inhibition of molecular pathways regulating cell lineage plasticity; however, specific treatment regimens and mechanisms still require further exploration [16].

For personalized treatment after transformation, epigenetics showed that transformed SCLC was more similar to SCLC than NSCLC based on their mRNA expressions and drug sensitivities [40]. Previous studies have also shown that chemotherapy, which is sensitive to classic SCLC (platinum and etoposide), may be the best treatment option for transformed SCLC, and taxanes also showed high response rates [41,42]. However, some transformed SCLC may partially retain the characteristics of NSCLC, and treatments for NSCLC and SCLC differ substantially. Despite the generally recognized SCLC-sensitive chemotherapy, treatment strategies should be decided after thorough considerations, and specific individualized treatments should be administered, considering the pathological and gene mutation results of re-biopsies. For instance, a SCLC-transformed patient with both exon 19 deletion and Thr790Met mutation received a combination of chemotherapy and osimertinib and achieved obvious clinical responses [43]. Another patient with an initial EGFR L858R mutation received alternating therapy of chemotherapy (carboplatin and irinotecan) and osimertinib for one year after SCLC transformation. The serum level of the tumor biomarkers showed that osimertinib led to a decrease in sialyl Lewis X (SLX), while the pro-GRP levels declined after administration of chemotherapy [44]. A 76-year-old female patient with EGFR L858R-mutant adenocarcinoma was diagnosed with SCLC transformation with consistent L858R mutation and received chemotherapy (cisplatin and etoposide) as the subsequent treatment for 5 months, after which she underwent both chemotherapy and immunotherapy (amrubicin, nivolumab, and irinotecan); however, further progression was observed after 6 months, and a second re-biopsy revealed both L858R and T790M mutations, which led to the administration of osimertinib, and a partial response was achieved and lasted for 3 months [45]. In addition, some of the SCLC-transformed patients experienced further gene mutations and histologic changes, and it was reported that NSCLC could be dominant again during treatment [46,47,48,49]. Therefore, treatment strategies should be selected according to the individual characteristics of patients. Considering that the histopathological types and gene mutations may change during the process of treatment for transformation, continuous monitoring and repeated re-biopsy are of significance for treatment decisions.

Moreover, clinical trials have shown that immune checkpoint inhibitors can improve the prognosis of patients with NSCLC and SCLC [50,51,52,53]. However, it has also been suggested that the application of EGFR-TKIs may reduce the expression of PD-L1 [9,54]. A previous retrospective study demonstrated that patients with transformed SCLC from EGFR-mutant adenocarcinomas had a poor response to checkpoint inhibitors [42]. Several cases have reported patients administered immunotherapy after SCLC transformation in clinical practice. Three cases reported patients with EGFR-mutant adenocarcinoma receiving immunotherapy (nivolumab) following other treatments and disease progression after SCLC transformation; however, immunotherapy did not show much efficacy [9,45,55]. Two patients with squamous cell NSCLC who were previously treated with nivolumab also received nivolumab after SCLC transformation; however, one patient experienced disease progression after 1 month, while the other was lost to follow-up [29]. A 62‑year‑old male patient with ALK rearrangement adenocarcinoma received subsequent combined immunotherapy and chemotherapy (amrubicin, irinotecan, and nivolumab) after SCLC transformation following targeted therapy and chemotherapy, which also showed a poor response [56]. A 75-year-old female patient with advanced KRAS G12C-mutant lung adenocarcinoma had previously received 33 cycles of nivolumab as second-line therapy. After SCLC transformation, she received chemotherapy (carboplatin and etoposide), immunotherapy (nivolumab and ipilimumab), and chemotherapy (irinotecan) in sequence and achieved a further 16-month survival [57]. Therefore, further investigations to determine whether immunotherapy or combined immunotherapy is promising for patients with SCLC transformation are recommended, and the levels of expression of PD-L1 should be monitored.

## 5 Conclusion

In conclusion, the transformation from NSCLC to SCLC should be further researched as a specific phenotype, as it has distinct characteristics from those of NSCLC and SCLC. Clinicians should be aware of the significance of re-biopsy and the dynamic monitoring of relevant biomarkers. Precision medicine is necessary for transformed patients based on individualized characteristics.
